# Clinicopathological Analysis of Ocular Adnexal Extranodal Marginal Zone B-Cell Lymphoma with IgG4-Positive Cells

**DOI:** 10.1371/journal.pone.0131458

**Published:** 2015-06-25

**Authors:** Min Joung Lee, Namju Kim, Ji-Young Choe, Sang In Khwarg, Yoon Kyung Jeon, Ho-Kyung Choung, Ji Eun Kim

**Affiliations:** 1 Department of Ophthalmology, Hallym University Sacred Heart Hospital, Anyang, Korea; 2 Department of Ophthalmology, Seoul National University Bundang Hospital, Seungnam, Korea; 3 Department of Pathology, Seoul National University Bundang Hospital, Seungnam, Korea; 4 Department of Ophthalmology, Seoul National University Hospital, Seoul, Korea; 5 Department of Pathology, Seoul National University Hospital, Seoul, Korea; 6 Department of Ophthalmology, Seoul National University Boramae Hospital, Seoul, Korea; 7 Department of Pathology, Seoul National University Boramae Hospital, Seoul, Korea; Istituto dei tumori Fondazione Pascale, ITALY

## Abstract

This study aims to analyze clinical and pathological characteristics of ocular adnexal extranodal marginal zone B-cell lymphoma (EMZL) accompanying IgG4-positive cells. Fifty patients with a diagnosis of primary non-conjunctival ocular adnexal EMZL were enrolled in this study. The number of IgG4-positive cells and the ratio of IgG/IgG4 were evaluated by immunohistochemistry in the biopsy specimens. The patients were divided into two groups based on the absolute number and the ratio of IgG4-positive cells (IgG4-posivite vs IgG4-negative groups). The demographic data, clinical staging at diagnosis, histopathological characteristics, and response to initial treatment were comparatively analyzed between the 2 groups. Five (10%) of 50 patients were defined as IgG4-positive group, and all the cases showed characteristic histological features such as extensive plasma cell infiltration and dense fibrosis. Most of these patients (4 of 5 patients) had lymphoma of the lacrimal gland. The patients from the IgG4-positive group showed a lower response rate to initial treatment (87.5 vs 33%, *p* = 0.03) than IgG4-negative group with a median follow-up period of 38 months. A part of the ocular adnexal EMZLs were accompanied with IgG4-positive cells. Significantly, most IgG4-positive ocular adnexal EMZLs occurred in the lacrimal gland, and can be related with a more frequent treatment failure.

## Introduction

Recognition of IgG4-related sclerosing disease is clinically important because it can be mistaken for malignancy owing to its tumefactive nature. Essentially, it is non-neoplastic disease that is histologically characterized by dense sclerosis and by the presence of abundant IgG4-positive plasma cells [1–3]. Individual organs, including the pancreas, bile duct, retroperitoneal soft tissues, liver, thyroid, lung, and salivary glands can be involved, or it can present as a systemic condition [2]. Although the prototype of IgG4-related disease in the ophthalmologic field is bilateral dacryoadenitis accompanied by sialadenitis (formerly known as Mikulicz disease), involvement of other ocular adnexal tissues such as orbital fat, extraocular muscles, and the lacrimal sac has been reported [4–6].

Several studies have investigated a potential relationship between IgG4-related disease and extranodal marginal zone B-cell lymphoma (EMZL) [7–10]. Cheuk et al initially described 3 cases of ocular adnexal lymphoma in patients with chronic IgG4-related sclerosing dacryoadenitis, and 3 cases of ocular adnexal EMZL displaying histological background of sclerosing inflammation and the presence of IgG4-positive plasma cells [9]. More recently, Kubota et al reported 10 cases of ocular adnexal EMZL accompanied by a heavy infiltration of IgG4-positive plasma cells out of 114 cases [7]. These results suggest that IgG4-positive plasma cells might participate in the pathogenesis of EMZL in a subset of the cases. However, clinical implications of this finding had not been fully investigated and further studies are needed to support these studies.

Therefore, the present study was designed to establish clinical and pathological characteristics of ocular adnexal EMZL accompanying IgG4-positive cells.

## Methods

### Patients selection

We surveyed our database for patients with a diagnosis of ocular adnexal EMZL, confirmed by a surgical biopsy at the Department of Ophthalmology of Seoul National University, Seoul National University Boramae Hospital, and Seoul National University Bundang Hospital between 2000 and 2010. Patients with solely conjunctival lymphoma or secondary lymphoma were excluded from the study. Clinical data were obtained from medical records, and pretreatment biopsy specimens were collected from all patients. The written informed consent was waived due to the study’s retrospective nature. This study followed the tenets of Helsinki Declaration, and the data were analyzed anonymously. The protocol for this study was approved by the institutional review board of Seoul National University Boramae Hospital (16-2013-19).

### Histopathological features of EMZL and IgG/IgG4 scoring

Serial 4-μm-thick sections were sliced from formalin-fixed and paraffin-embedded tissue blocks, and subjected to haematoxylin and eosin (H&E) staining and immunohistochemistry. Three haematopathologists (J.E.K, Y.K.J., and J.Y.C) reviewed all H&E and immunohistochemistry slides and achieve a consensus. PCR-based gene rearrangement study for immunoglobulin heavy chain was performed for a few selected cases. Immunohistochemical staining of paraffin-embedded tissue sections was performed as previously described.^11^ Each 4-μm tissue section was incubated with anti-IgG (1:10000, DakoCytomation, Copenhagen, Denmark) and anti-IgG4 (1:5000, Invitrogen, Carlsbad, CA, USA), using a BenchMark automatic immunostaining device (Ventana Medical Systems, Tucson, AZ, USA) with standard heat-induced antigen retrieval.

The extent of IgG4-positive plasma cell infiltration was evaluated either by the absolute number of IgG4-positive cells or by the ratio of IgG4 to IgG-positive cells per high-power field (HPF), defined as a region with the highest density of immunostained cells, covering an area of 0.14 mm^2^. Images were taken by a DP72 digital camera mounted on a BX51 microscope (Olympus Corp., Tokyo, Japan). The patients with IgG4/IgG cell ratio > 40% and IgG4-positive plasma cell density ≥ 50/HPF were classified as IgG4-positive ocular adnexal EMZL patients (IgG4-positive group). The patients who did not meet the requirements were considered as IgG4-negative ocular adnexal EMZL (IgG4-negative group) [2].

### Detection of *Chlamydia psittaci*

Presence of *Chlamydia psittaci* was tested in 32 samples of ocular adnexal EMZL via touchdown enzyme time-release PCR (TETR-PCR) as previously described [11]. Genomic DNA was extracted from paraffin-embedded tumor tissues using QIAamp DNA FFPE kit (Qiagen, Valencia, CA, USA). The primer sequences for *Cp* were 5'-CCC AAG GTG AGG CTG ATG AC-3' (forward) and 5'-CAA ACC GTC CTA AGA CAG TTA-3' (reverse). Ta-CLONED *Cp* DNA was used as a positive control. The annealing temperature was 54°C. The amplified DNA fragments were electrophoresed on 2% agarose gels and were visualized after staining with ethidium bromide.

### Clinical data

Disease stage was defined according to the American Joint Committee on Cancer (AJCC) classification [12]. The response rate and progression-free survival to initial treatment were also compared in patients who were followed up more than 6 months. Response assessments were established according to the revised response criteria for malignant lymphoma defined as follows: complete remission (CR), complete disappearance of all clinical evidence of lymphoma based on physical examination and orbital computed tomography or magnetic resonance imaging; partial remission (PR), regression of measurable disease and no new disease sites; relapsed or progressive disease (PD), any new lesion or increase in size by ≥50% from the nadir at previously involved sites; and stable disease (SD), failure to attain CR,PR or PD [13].

Progression-free survival was calculated from the date of first-line treatment to the date of disease progression, death, or the last follow-up visit. The Fisher’s exact test was used to compare possible associations between the categorical variables. For the continuous variables, the Mann-Whitney U test was used. For all the tests, *p* < 0.05 was considered to be statistically significant. All the statistical analyses were performed using the SPSS software (SPSS for windows Release, version 14.0, SPSS, Chicago, IL, USA).

## Results

**Fig 1:**
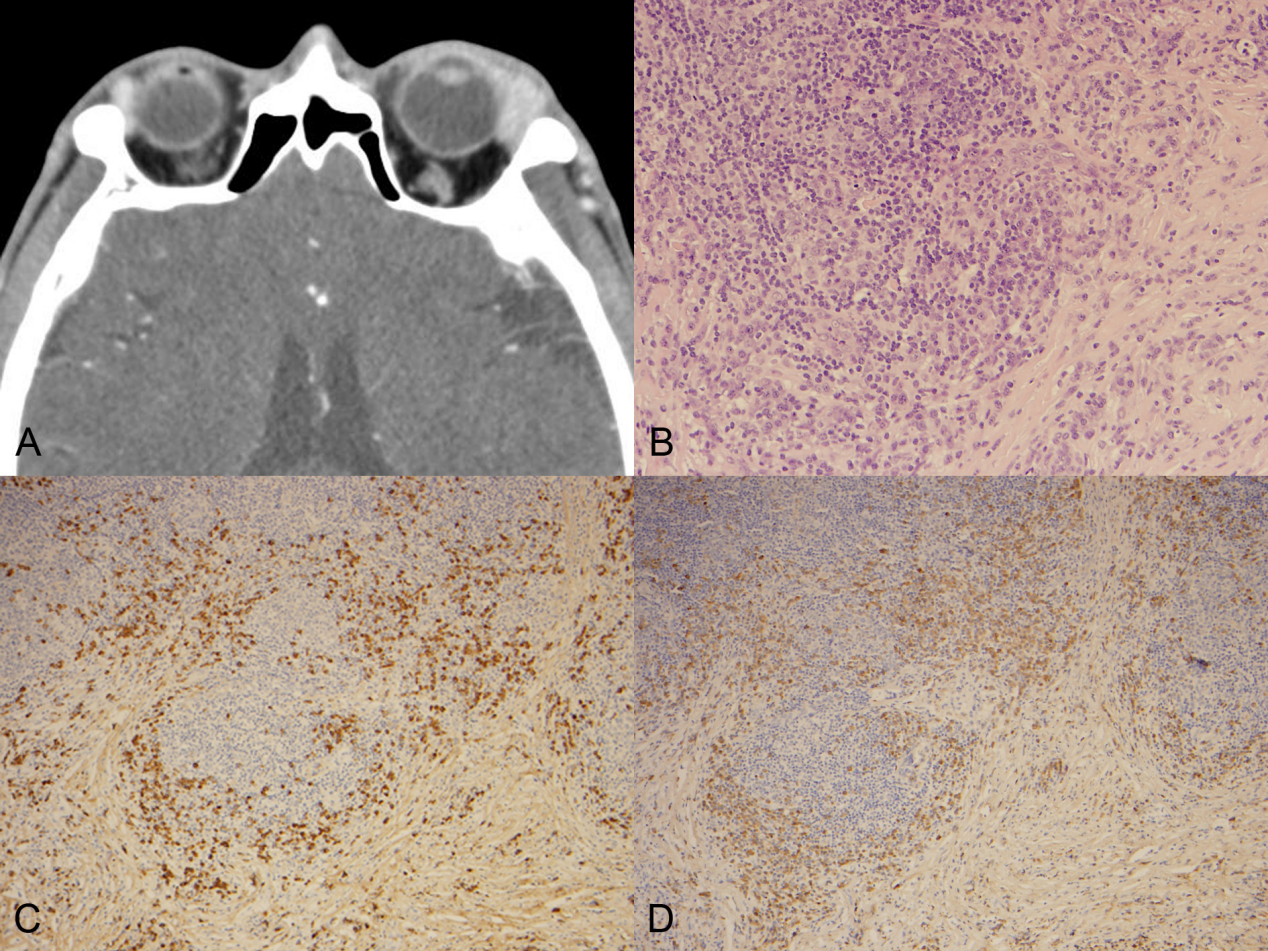
(A) Computed tomographic image revealing an enlarged lacrimal gland of the left side, with homogenous enhancement. (B) Histological examination result showing lymphoplasma cell infiltration and background fibrosis (H&E, original magnification ×200). Immunohistochemical staining for (C) IgG and (D) IgG4 (original magnification ×200) showing that most of the IgG-positive cells are positive for IgG4.

Data from 143 patients with ocular adnexal EMZL were retrieved from medical records by computerized search. Among them, 88 patients with conjunctival lymphoma and 5 patients without available tissue-blocks were excluded, and finally 50 patients were enrolled in this study. Ten of fifty patients were overlapped with previous research conducted at Seoul National University Hospital [14]. The mean age was 56 years (range: 23–72 years) with a male-female ratio of 35:15, and bilateral involvement was observed in 8 patients (16.0%). The most common presenting symptom was palpable mass in 17 patients (34%), followed by eyelid swelling (13 patients, 26%), proptosis (11 patient, 22%), diplopia (4 patients, 8%), ptosis (3 patients, 6%), and decreased vision (2 patients, 4%). None of the patients complained B symptoms. Serum LDH values were available for 40 patients and 8 patients (20%) showed elevated LDH levels. All cases diagnosed with ocular adnexal EMZL showed characteristic histologic features such as dense small lymphoid cell infiltration with only slight atypia, variable degree of plasmacytoid differentiation, invasive features represented by follicular colonization or lymphoepithelial lesions. Immunohistochemical stain revealed that tumor cells were diffusely positive for CD20, CD79a, bcl-2, and negative for CD3, CD5, CD10, cyclin D1 and bcl-6, which excludes the possibility of low grade B-cell lymphoma other than EMZL. Based on IgG and IgG4 immunostaining, 5 patients (10%) were defined as the IgG4-positive group and 45 patients were considered as the IgG4-negative group. Histologically, all the 5 patients in the IgG4-positive group showed dense plasma cell infiltration and fibrosis, and lymphoid follicle formation was observed in 3 patients (Fig 1).

The demographic data including age, gender, and clinical stage at diagnosis were collected and compared between the IgG4-positive and IgG4-negative groups (Table 1). Age, gender, laterality, and duration of symptom were not significantly different between the 2 groups. As determined by AJCC staging, 5 patients in the IgG4-positive group had orbital lymphoma (T2) and the IgG4-negative group consisted of 41 patients with orbital lesion (T2), 3 patients with eyelid lesion (T3), and 1 patient with a mass extending to the cheek (T4). For the patients with orbital lymphoma (T2), a subgroup analysis was performed, and significant differences were found between the 2 groups (*p* = 0.02). In the IgG4-positive group, most of the patients (4 of 5 patients) had lacrimal gland lymphoma (T2b), whereas most of the patients in the IgG4-negative group (25 of 41 patients) showed lymphoma occupying the posterior orbit (T2c). Although no patient showed lymph node metastasis, 3 patients in the IgG4-negative group presented distant metastasis at diagnosis. *Cp* DNA was observed in 16 (50%) of 32 patients with available samples. *Cp*-positivity was 75.0% (3 of 4) in IgG4-positive group and 46% (13 of 28) in IgG4-negative group. However, there was no statistically significant difference between two groups (*p* = 0.59).

**Table 1:** Characteristics of ocular adnexal extranodal marginal zone B-cell lymphoma (EMZL) patients according to the IgG4-positive plasma cell infiltration.

Variables	IgG4-positive group (n = 5)	IgG4-negative group (n = 45)	*p*
**Gender (Male: Female)**	4:1	31:14	1.00*
**Age at diagnosis (mean, years)**	53.4 ± 14.9 (range 39–72)	56.0 ± 9.4 (range 23–76)	0.58^†^
**Laterality (Bilateral: Unilateral)**	1: 4	7:38	1.00*
**Duration of symptom (mean, months)**	30.0 ± 50.6	17.9 ± 22.8	0.33^†^
**Clinical staging by AJCC**			
Primary tumor (T2:T3:T4)	5:0:0	41:3:1	1.00*
*T2a*:*T2b*:*T2c*:*T2d*	0:4:1:0	8:5:25:3	0.02*
Lymph node involvement (N)	0	0	-
Distant metastasis (M)	0	3	1.00*
**LDH (Normal: Elevated)**	4:1 (n = 5)	33:7 (n = 40)	1.00*
**IPI (Low:Low-intermediate)**	5:0 (n = 5)	35:5 (n = 40)	1.00*
***Chlamydia psittaci* DNA**			
Positive: Negative	3:1 (n = 4)	13:15 (n = 28)	0.59*

*Fisher’s exact test.

^†^Mann-Whitney U test.

IgG4-positive group, ocular adenexal EMZL with IgG4:IgG cell ratio > 40%, and IgG4-positive plasma cells ≥ 50 / HPF; IgG4-negative group, ocular adenexal EMZL with IgG4-positive plasma cells < 50 / HPF or IgG4:IgG cell ratio ≤ 40%; AJCC, American Joint Committee on Cancer classification; LDH, Lactate dehydrogenase; IPI, International Prognostic Index.

Forty-three patients (3 patients in the IgG4-positive group and 40 patients in the IgG4-negative group) were followed up for more than 6 months and were therefore eligible for the treatment outcome analysis (Table 2). Front-line treatment was administered as follows: combination chemotherapy in 29 patients, doxycycline treatment in 8 patients, and radiation therapy in 6 patients. With a median follow-up period of 38 months, the IgG4-negative group showed better treatment response than the IgG4-positive group (*p* = 0.03). The IgG4-negative group showed prolonged progression-free survival compared with the IgG4-positive group, although the results did not reach statistical significance (33 vs 24 months, *p* = 0.08, log-rank test).

**Table 2:** Treatment outcome in ocular adnexal extranodal marginal zone B-cell lymphoma (EMZL) patients according to the IgG4-positive plasma cell infiltration.

	IgG4-positive group(n = 3)	IgG4-negative group(n = 40)	*p*
**Follow-up duration (median, months)**	56.0 ± 29.1	42.6 ± 32.5	1.00^†^
**Initial treatment modalities**			
Radiation	1 patient	5 patients	
Chemotherapy	2 patients	27 patients	
Doxycycline	-	8 patients	
**Response to treatment (CR:PR:SD:PD)**	1:0:2:0	14:21:5:0	0.03*
**Response rate**	33%	87.5%	0.06*
**Response to chemotherapy (CR:PR:SD:PD)**	0:0:2:0	12:14:1:0	0.007*

*Fisher’s exact test.

^†^Mann-Whitney U test.

IgG4-positive group, ocular adenexal EMZL with IgG4:IgG cell ratio > 40%, and IgG4-positive plasma cells ≥ 50 / HPF; IgG4-negative group, ocular adenexal EMZL with IgG4-positive plasma cells < 50 / HPF or IgG4:IgG cell ratio ≤ 40%.

CR, complete response; PD, progressive disease; PR, partial response; SD, stable disease.

## Discussion

IgG4-related disease is a recently proposed entity with several unique clinicopathological features, such as enlargement of affected organs, elevated serum IgG4 level, and infiltration with IgG4-positive plasma cells [1,3,15]. IgG4-related disease is a conceptually systemic and chronic inflammatory disorder, and patients show various symptoms according to the affected organs. Corticosteroid therapy has been shown to be effective in these patients, but disease relapse occurs frequently. Although IgG4-related disease is getting to form a distinct clinical entity, many questions and problems remain to be elucidated, including its association with malignant lymphoma. In this study, 5 of 50 ocular adnexal EMZL cases were categorized as IgG4-positive, and were predominantly located at the lacrimal gland. This group was also associated with a lower response rate to initial treatment compared with the IgG4-negative group, although the number of cases was small.

Recently, Kubota et al analyzed 114 biopsy samples of ocular adnexal EMZLs, and reported IgG4-positive plasma cell infiltration in 9% of cases, similar to the results of the present study (10%) [7]. However, the eligible criteria of patients were somewhat different between the two studies. Many previous studies revealed a variable proportion of IgG4-related lesions among ocular adnexal EMZLs because of the lack of consensus on the criteria defining IgG4-related disease. In contrast to the study by Kubota et al, we used both the absolute number of IgG4-positive cells and the ratio of IgG4/IgG-positive cells as criteria for grouping in addition to the ratio for defining. In addition, we excluded conjunctival EMZL (T1) because it is known that IgG4-related disease rarely involves conjunctiva [14]. Sato et al reported that the conjunctiva was not involved in any of the 21 patients with ocular adnexal IgG4-related disease in their study [1]. We find only one case report on IgG4-related EMZL involving the conjunctiva through extensive literature search [16].

IgG4-positive group showed lacrimal gland preponderance either unilaterally or bilaterally (T2b, 4 of 5 cases), whereas the IgG4-negative group revealed an even distribution of the primary site throughout the orbital tissues [7]. Our result is also in agreement with the previous study reporting 6 cases of IgG4-related sclerosing dacryoadenitis associated with EMZL of the lacrimal gland [9]. Indeed, along with the pancreas, the lacrimal gland is known as a main target organ of IgG4-related disease [3,15]. However, there is a controversial finding by Kubota et al who reported higher number of IgG4-positive EMZL of the posterior orbit (8 cases) rather than the lacrimal gland (2 cases) [7].

A link between ocular adnexal IgG4-related disease and EMZL has been proposed [17,18]. EMZL can be caused by chronic infection or inflammation [19–21]. Patients with autoimmune diseases, such as Sjogren’s syndrome or Hashimoto’s thyroiditis are at increased risk of developing EMZLs [19]. However, the causal relationship between IgG4-related disease and EMZL remains uncertain, as IgG4-positive lymphomas can develop independently or arise from underlying IgG4-related disease. In this study, 3 of 5 patients in the IgG4-positive group had some clinical features that could be associated with systemic IgG4-related disease (Table 3). Case 1 had a history of dacryoadenitis in the contralateral lacrimal gland, and case 2 had a history of bilateral sialadenitis. Although serum IgG4 data or pathological reports of these lesions are unavailable, based on the clinical history, it is highly likely that the 2 cases are associated with systemic IgG4-related disease. It is typical for IgG4-related sclerosing disease that the lacrimal and salivary glands are involved either synchronously or metachronously, suggesting that IgG4-related disease might precede EMZL [3]. Another interesting feature is found in case 3 which had asthma. Allergic features, such as asthma, atopic dermatitis, eczema and peripheral eosinophilia are also frequently accompanied by IgG4-related disease. In a study of 9 patients with IgG4-related lacrimal gland enlargement, elevated serum IgE levels were found in all patients, and 5 of 9 patients exhibited eosinophilia and asthma-like symptoms, suggesting that a Th2 cell-mediated allergic response may be involved in the pathogenesis of IgG4-related disease [22].

**Table 3:** Clinicopathologic features of ocular adnexal extranodal marginal zone B-cell lymphoma (EMZL) patients with of IgG4-positive plasma cell infiltration.

	Case 1	Case 2	Case 3	Case 4	Case 5
Age / Gender	45 / M	67 / M	39 / F	44 / M	72 / M
Presenting symptom	Left upper eyelid swelling	Left upper eyelid mass	Both upper eyelid mass	Right upper eyelid swelling	Diplopia
Duration of symptom	9	12	12	3	3
Tumor location	Left lacrimal gland	Left lacrimal gland	Both lacrimal gland	Right lacrimal gland	Right superonasal orbit
TNM staging	T2bN0M0	T2bN0M0	T2bN0M0	T2bN0M0	T2cN0M0
No. of IgG4(+) cells/HPF	151 ± 44	268 ± 53	122 ± 42	96 ± 30	110 ± 40
IgG4(+) /IgG(+) cells (%)	60	77	60	60	40
LF / sclerosis / eosinophils	+ /—/ +	+ / + /-	+ / +/+	- / + /-	- / + / -
*Chlamydia psittaci* DNA	Positive	Positive	Negative	Positive	Not available
Follow-up period (months)	24	56	82	2	1
Initial treatment modality	Chemotherapy	Chemotherapy	Radiation	-	-
Response to treatment (CR:PR:SD:PD)	SD	SD	CR	-	-
Clinical remarks	Contralateral orbital pseudotumor	Nonspecific sialadenitis	Asthma	-	-

*Proposed by American Joint Committee on Cancer^12^

LF, lymphoid follicle; CR, complete response; PD, progressive disease; PR, partial response; SD, stable disease.

Regarding pathogenesis of ocular adnexal EMZL, several genetic abnormalities including chromosomal aneuploidy and gene translocations affecting activation of NF-Kb pathway have been reported [23]. Unlike gastric MALT lymphoma, these genetic alterations occur randomly in a minority of the cases, moreover, are not sufficient enough for malignant transformation in ocular adnexal EMZL [24]. Association with *Cp* has also been reported [19,25]. The prevalence of *Cp* was known to have geographical difference, and some studies from Korea reported relatively high prevalence over 70% [23,26,27]. We also found frequent association of *Cp* in this study, showing similar incidence reported by other Korean researchers. Although *Cp*-positivity was slightly higher (75%) in IgG4-positive group, than IgG4-negative group (46%), the difference was not fully significant because of the small number of IgG4-positive cases. One possible suggestion is that *Cp* infection is just prevalent in longstanding inflammatory lesions of ocular area in our country, not directly associated with recruitment of IgG4+ cells. Further investigations will be needed to clarify this issue.

There are no established prognostic indicators for the treatment of ocular adnexal EMZL, although CD5+, trisomy 18, and high Ki-67 have been shown to correlate with a poor outcome in some patients [28–30]. Recently, Bi et al reported that A20 inactivation by mutation of deletion in ocular adnexal EMZL was associated with increased expression of NF-Kb target genes, and required higher radiation dosages to achieve complete remission [31]. In this study, IgG4-negative group showed significantly better response to front-line treatment than IgG4-positive group. There has been no literature of the clinical usefulness of IgG4-positivity as a predictor. We suggested that IgG4 may be a possible prognostic indicator in ocular adnexal EMZL. However, large scale studies should be performed to confirm our finding.

Because of its retrospective nature, there is no data about serum IgG4/IgG, which is one of the main limitations of this study. Although serum IgG4 level elevation is helpful in diagnosing IgG4-related disease, it is not a specific diagnostic marker. Rather than serum IgG4 concentration, histopathological findings, such as dense lymphoplasmacytic infiltration and storiform fibrosis, are more essential for diagnosis [32,33]. Recently proposed diagnostic criteria defined IgG4-related disease in patients with (1) organ enlargement, mass or nodular lesions, or organ dysfunction; (2) a serum IgG4 concentration > 135 mg/dL; and (3) histopathological findings > 10 IgG4 cells/HPF and an IgG4+/IgG+ cell ratio > 40%. However, IgG4+/IgG+ plasma cell ratio is considered as a more powerful value than IgG4+ cell count and the cutoff point for the IgG4+ plasma cell count may vary from organ to organ [34]. The proposed cut-offs for lacrimal lesions range from 10 to 100 IgG4+ cell/HPF[35].

In conclusion, some nonconjunctival ocular adnexal EMZLs were associated with IgG4-positive sclerosing disease. The patients in the IgG4-positive group showed masses predominantly in the lacrimal gland and had a poor response to initial therapy. Although the results from this study were limited to be generalized because of the small number of cases, our data suggest that ocular adnexal EMZL accompanying IgG4-positive cells has unique clinical features discriminated with those without IgG4-positive cells. Further large-scaled studies will be needed to confirm these findings.

## References

[1] Sato Y, Ohshima K, Ichimura K, Sato M, Yamadori I, Tanaka T, et al Ocular adnexal IgG4-related disease has uniform clinicopathology. Pathol Int. 2008;58(8):465–70. 10.1111/j.1440-1827.2008.02257.x . 18705764

[2] Umehara H, Okazaki K, Masaki Y, Kawano M, Yamamoto M, Saeki T, et al Comprehensive diagnostic criteria for IgG4-related disease (IgG4-RD), 2011. Mod Rheumatol. 2012;22(1):21–30. 10.1007/s10165-011-0571-z . 22218969

[3] Stone JH, Zen Y, Deshpande V. IgG4-related disease. N Engl J Med. 2012;366(6):539–51. 10.1056/NEJMra1104650 . 22316447

[4] Batra R, Mudhar HS, Sandramouli S. A Unique Case of IgG4 Sclerosing Dacryocystitis. Ophthal Plast Reconstr Surg. 2012;28(3):e70–2. 10.1097/IOP.0b013e31822d7f9b . 21946772

[5] Pasquali T, Schoenfield L, Spalding SJ, Singh AD. Orbital inflammation in IgG4-related sclerosing disease. Orbit. 2011;30(5):258–60. 10.3109/01676830.2011.593677 . 21957960

[6] Wallace ZS, Khosroshahi A, Jakobiec FA, Deshpande V, Hatton MP, Ritter J, et al IgG4-related systemic disease as a cause of "idiopathic" orbital inflammation, including orbital myositis, and trigeminal nerve involvement. Surv Ophthalmol. 2012;57(1):26–33. 10.1016/j.survophthal.2011.07.004 . 22018678

[7] Kubota T, Moritani S, Yoshino T, Nagai H, Terasaki H. Ocular adnexal marginal zone B cell lymphoma infiltrated by IgG4-positive plasma cells. J Clin Pathol. 2010;63(12):1059–65. 10.1136/jcp.2010.082156 20980530 PMC2991078

[8] Oyama T, Takizawa J, Nakamura N, Aoki S, Aizawa Y, Abe H. Multifocal mucosa-associated lymphoid tissue lymphoma associated with IgG4-related disease: a case report. Jpn J Ophthalmol. 2011;55(3):304–6. 10.1007/s10384-011-0003-9 . 21584726

[9] Cheuk W, Yuen HK, Chan AC, Shih LY, Kuo TT, Ma MW, et al Ocular adnexal lymphoma associated with IgG4+ chronic sclerosing dacryoadenitis: a previously undescribed complication of IgG4-related sclerosing disease. Am J Surg Pathol. 2008;32(8):1159–67. 10.1097/PAS.0b013e31816148ad . 18580683

[10] Kubota T, Moritani S, Yoshino T, Nagai H, Terasaki H. Ocular adnexal mucosa-associated lymphoid tissue lymphoma with polyclonal hypergammaglobulinemia. Am J Ophthalmol. 2008;145(6):1002–6. 10.1016/j.ajo.2008.01.006 . 18336788

[11] Choung HK, Kim YA, Lee MJ, Kim N, Khwarg SI. Multigene methylation analysis of ocular adnexal MALT lymphoma and their relationship to Chlamydophila psittaci infection and clinical characteristics in South Korea. Invest Ophthalmol Vis Sci. 2012;53(4):1928–35. 10.1167/iovs.11-7668 . 22410569

[12] Coupland SE, White VA, Rootman J, Damato B, Finger PT. A TNM-based clinical staging system of ocular adnexal lymphomas. Arch Pathol Lab Med. 2009;133(8):1262–7. 10.1043/1543-2165-133.8.1262 . 19653722

[13] Cheson BD, Pfistner B, Juweid ME, Gascoyne RD, Specht L, Horning SJ, et al Revised response criteria for malignant lymphoma. J Clin Oncol. 2007;25(5):579–86. 10.1200/jco.2006.09.2403 . 17242396

[14] Go H, Kim JE, Kim YA, Chung HK, Khwarg SI, Kim CW, et al Ocular adnexal IgG4-related disease: comparative analysis with mucosa-associated lymphoid tissue lymphoma and other chronic inflammatory conditions. Histopathology. 2012;60(2):296–312. 10.1111/j.1365-2559.2011.04089.x . 22211288

[15] Masaki Y, Kurose N, Umehara H. IgG4-related disease: a novel lymphoproliferative disorder discovered and established in Japan in the 21st century. J Clin Exp Hematop. 2011;51(1):13–20. . 21628856 10.3960/jslrt.51.13

[16] Paulus YM, Cockerham KP, Cockerham GC, Gratzinger D. IgG4-positive sclerosing orbital inflammation involving the conjunctiva: a case report. Ocular immunology and inflammation. 2012;20(5):375–7. 10.3109/09273948.2012.709574 . 23030356

[17] Yamamoto M, Takahashi H, Shinomura Y. IgG4-Related Disease and Malignancy. Intern Med. 2012;51(4):349–50. . 22333367 10.2169/internalmedicine.51.6782

[18] Yamamoto M, Takahashi H, Tabeya T, Suzuki C, Naishiro Y, Ishigami K, et al. Risk of malignancies in IgG4-related disease. Mod Rheumatol. 2011. 10.1007/s10165-011-0520-x . 21894525

[19] Sagaert X, De Wolf-Peeters C, Noels H, Baens M. The pathogenesis of MALT lymphomas: where do we stand? Leukemia. 2007;21(3):389–96. 10.1038/sj.leu.2404517 . 17230229

[20] Sagaert X, Van Cutsem E, De Hertogh G, Geboes K, Tousseyn T. Gastric MALT lymphoma: a model of chronic inflammation-induced tumor development. Nat Rev Gastroenterol Hepatol. 2010;7(6):336–46. 10.1038/nrgastro.2010.58 . 20440281

[21] van de Schans SA, van Spronsen DJ, Hooijkaas H, Janssen-Heijnen ML, Coebergh JW. Excess of autoimmune and chronic inflammatory disorders in patients with lymphoma compared with all cancer patients: a cancer registry-based analysis in the south of the Netherlands. Autoimmun Rev. 2011;10(4):228–34. 10.1016/j.autrev.2010.11.001 . 21074639

[22] Kanari H, Kagami S, Kashiwakuma D, Oya Y, Furuta S, Ikeda K, et al Role of Th2 cells in IgG4-related lacrimal gland enlargement. Int Arch Allergy Immunol. 2010;152(suppl 1):47–53. 10.1159/000312125 . 20523063

[23] Collina F, De Chiara A, De Renzo A, De Rosa G, Botti G, Franco R. Chlamydia psittaci in ocular adnexa MALT lymphoma: a possible role in lymphomagenesis and a different geographical distribution. Infectious agents and cancer. 2012;7:8 10.1186/1750-9378-7-8 . 22472082 PMC3355003

[24] Stefanovic A, Lossos IS. Extranodal marginal zone lymphoma of the ocular adnexa. Blood. 2009;114(3):501–10. 10.1182/blood-2008-12-195453 . 19372259 PMC2713468

[25] Ferreri AJ, Dolcetti R, Du MQ, Doglioni C, Resti AG, Politi LS, et al Ocular adnexal MALT lymphoma: an intriguing model for antigen-driven lymphomagenesis and microbial-targeted therapy. Ann Oncol. 2008;19(5):835–46. 10.1093/annonc/mdm513 . 17986622

[26] Yoo C, Ryu MH, Huh J, Park JH, Kang HJ, Ahn HS, et al Chlamydia psittaci infection and clinicopathologic analysis of ocular adnexal lymphomas in Korea. American journal of hematology. 2007;82(9):821–3. 10.1002/ajh.20962 . 17570512

[27] Kim TM, Kim KH, Lee MJ, Jeon YK, Lee SH, Kim DW, et al First-line therapy with doxycycline in ocular adnexal mucosa-associated lymphoid tissue lymphoma: a retrospective analysis of clinical predictors. Cancer Sci. 2010;101(5):1199–203. 10.1111/j.1349-7006.2010.01502.x . 20345477 PMC11158853

[28] Coupland SE, Hellmich M, Auw-Haedrich C, Lee WR, Stein H. Prognostic value of cell-cycle markers in ocular adnexal lymphoma: an assessment of 230 cases. Graefes Arch Clin Exp Ophthalmol. 2004;242(2):130–45. 10.1007/s00417-003-0831-5 . 14685876

[29] Ferry JA, Yang WI, Zukerberg LR, Wotherspoon AC, Arnold A, Harris NL. CD5+ extranodal marginal zone B-cell (MALT) lymphoma. A low grade neoplasm with a propensity for bone marrow involvement and relapse. Am J Clin Pathol. 1996;105(1):31–7. . 8561085 10.1093/ajcp/105.1.31

[30] Gruenberger B, Woehrer S, Troch M, Hauff W, Lukas J, Streubel B, et al Assessment of the role of hepatitis C, Helicobacter pylori and autoimmunity in MALT lymphoma of the ocular adnexa in 45 Austrian patients. Acta Oncol. 2008;47(3):355–9. 10.1080/02841860701630283 . 17957504

[31] Bi Y, Zeng N, Chanudet E, Huang Y, Hamoudi RA, Liu H, et al A20 inactivation in ocular adnexal MALT lymphoma. Haematologica. 2012;97(6):926–30. 10.3324/haematol.2010.036798 . 22207688 PMC3366661

[32] Sah RP, Chari ST. Serologic issues in IgG4-related systemic disease and autoimmune pancreatitis. Curr Opin Rheumatol. 2011;23(1):108–13. 10.1097/BOR.0b013e3283413469 . 21124093

[33] Strehl JD, Hartmann A, Agaimy A. Numerous IgG4-positive plasma cells are ubiquitous in diverse localised non-specific chronic inflammatory conditions and need to be distinguished from IgG4-related systemic disorders. J Clin Pathol. 2011;64(3):237–43. 10.1136/jcp.2010.085613 . 21233087

[34] Deshpande V, Zen Y, Chan JK, Yi EE, Sato Y, Yoshino T, et al Consensus statement on the pathology of IgG4-related disease. Modern pathology: an official journal of the United States and Canadian Academy of Pathology, Inc. 2012;25(9):1181–92. 10.1038/modpathol.2012.72 . 22596100

[35] Andrew N, Kearney D, Selva D. IgG4-related orbital disease: a meta-analysis and review. Acta ophthalmologica. 2013;91(8):694–700. 10.1111/j.1755-3768.2012.02526.x . 22963447

